# Recent advances of bispecific antibodies in solid tumors

**DOI:** 10.1186/s13045-017-0522-z

**Published:** 2017-09-20

**Authors:** Shengnan Yu, Anping Li, Qian Liu, Xun Yuan, Hanxiao Xu, Dechao Jiao, Richard G. Pestell, Xinwei Han, Kongming Wu

**Affiliations:** 1 Department of Oncology, Tongji Hospital of Tongji Medical College, Huazhong University of Science and Technology, 1095 Jiefang Avenue, Wuhan, 430030 China; 2 Department of Interventional Radiology, First Affiliated Hospital of Zhengzhou University, Zhengzhou, 450052 China; 3 Pennsylvania Center for Cancer and Regenerative Medicine, Wynnewood, PA 19096 USA

**Keywords:** BsAb, Solid tumor, EpCAM, CEA, PSMA, HER family, Radioimmunotherapy

## Abstract

Cancer immunotherapy is the most exciting advancement in cancer therapy. Similar to immune checkpoint blockade and chimeric antigen receptor T cell (CAR-T), bispecific antibody (BsAb) is attracting more and more attention as a novel strategy of antitumor immunotherapy. BsAb not only offers an effective linkage between therapeutics (e.g., immune effector cells, radionuclides) and targets (e.g., tumor cells) but also simultaneously blocks two different oncogenic mediators. In recent decades, a variety of BsAb formats have been generated. According to the structure of Fc domain, BsAb can be classified into two types: IgG-like format and Fc-free format. Among these formats, bispecific T cell engagers (BiTEs) and triomabs are commonly investigated. BsAb has achieved an exciting breakthrough in hematological malignancies and promising outcome in solid tumor as showed in various clinical trials. In this review, we focus on the preclinical experiments and clinical studies of epithelial cell adhesion molecule (EpCAM), human epidermal growth factor receptor (HER) family, carcinoembryonic antigen (CEA), and prostate-specific membrane antigen (PSMA) related BsAbs in solid tumors, as well as discuss the challenges and corresponding approaches in clinical application.

## Background

Although great progress has been achieved in the treatment for cancer, it is still difficult to be cured due to tumor recurrence, drug resistance, etc. [1]. Therefore, there is a critical need for the development of new treatment for those refractory or recurrent patients. Compared with other conventional therapeutic approaches, immunotherapy has a specific advantage [2]. Monoclonal antibody (mAb), tumor vaccine, immune checkpoint blockade [3–5], and most recently CAR-T and bispecific antibody (BsAb) are powerful tools for the immunologic treatment of cancer [6–8].

**Fig. 1:**
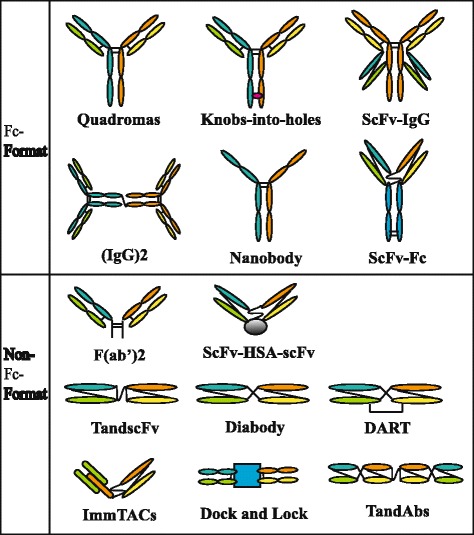
Molecular formats of bispecific antibodies. According to the Fc domain, BsAbs can be divided into two types: IgG-format molecules and non-IgG-format molecules. IgG-like BsAbs mainly include quandroma, knobs-into-holes, scFv-IgG, (IgG)2, scFv-Fc, and nanobody. The Fc-free BsAbs contain tandscFv, DART, TandAb, F(ab’)2, diabody, ImmTAC, Dock and Lock, and scFv-HSA-scF

In the mid-1980s, the BsAb was proposed to the treatment of cancers. Until recently, BsAb is intensively investigated [9]. BsAb can enhance tumor killing in a non-MHC-restricted manner by redirecting effector cells (e.g., T cells, NK cells, macrophages, and monocytes) to the tumor cells [10, 11]. Moreover, BsAb not only offers an effective linkage between therapeutics (e.g., immune effector cells, radionuclides) and targets (e.g., tumor cells) but also simultaneously blocks two different oncogenic mediators such as anti-epidermal growth factor receptor (EGFR) × anti-HER2 and anti- EGFR × anti-c-MET [12, 13]. With the development of advanced technology, many different BsAb formats have been proposed. According to the Fc domain, BsAb can be classified into two types: IgG-format and non-IgG-format [14]. The IgG-like molecules containing Fc domain retain Fc-mediated effector functions such as antibody-dependent cell mediated cytotoxicity (ADCC), complement-dependent cytotoxicity (CDC), and antibody-dependent cellular phagocytosis (ADCP) [15], mainly including quandroma, knobs-into-holes, scFv-IgG, and (IgG)2. The Fc-free BsAbs include TandscFv, DART, TandAb, F(ab’)2, Diabody, and ImmTAC [14] (Fig. 1). Triomabs and BiTEs are the most advanced BsAb formats among various BsAb molecules [16]. BiTEs are fusion proteins consisting of two single-chain variable fragments (scFv) connected by a short peptide linker. One of the scFvs binds to CD3 on T cells and the other to a surface antigen on tumor cells [17]. Blinatumomab, as a BiTE antibody against CD19/CD3, has been approved for the treatment of relapsed/refractory B-precursor acute lymphoblastic leukemia (ALL) by the US Food and Drug Administration (FDA) in December 2014 [18]. Due to the absence of Fc domain, BiTEs showed short serum half-lives, which hamper its clinical application. The permeability is an important issue in the treatment of solid tumors, and the permeability of BiTEs is greater than triomabs due to its small molecular mass (55KDa) [19]. Triomabs, as an IgG-like molecule, could bind two different tumor antigens simultaneously and interact with the FcR expressed on NK cells, macrophages, and dendritic cells through the Fc domain [20]. Because of the existence of Fc domain, triomabs show slower clearance from the blood than BiTEs. Nevertheless, the strong immunogenicity and compromised permeability of triomabs are just caused by Fc domain [21]. In 2009, catumaxomab, a triomab co-targeting EpCAM/CD3, was approved for the intraperitoneal treatment of malignant ascites in patients with EpCAM-positive cancers [22].

**Fig. 2:**
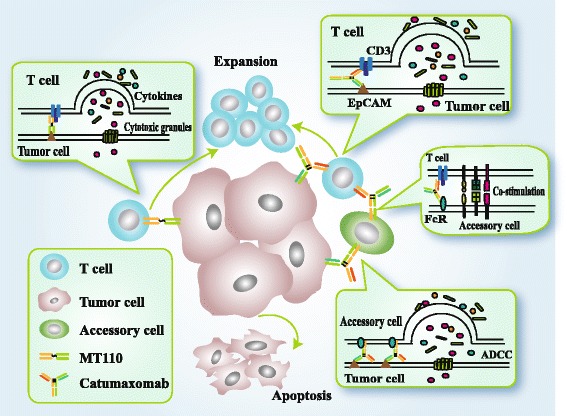
The comparison of BiTEs (taking MT110 as an example) and triomabs (taking catumaxomab as an example) killing mechanism. The interaction of T cells and tumor cells, which is mediated by BsAbs, initiates the killing process of T cells, including CD3 activation, formation of immunologic synapses, T-cell activation and proliferation, secretion of cytokines and cytotoxic granules, and tumor cell lysis [19]. The activated CD8^+^ and CD4^+^ T cells lyse cancer cells predominantly through perforin and granzyme B. The activated T cells secrete various cytokines such as IFN-γ, TNF, IL-2, IL-6, and IL-10 [20]. In addition to the above mechanism, triomabs can recruit other immune cells such as NK cells, macrophages which could kill tumor cells and mediate the co-stimulation between T cells and accessory cells [16]. Simultaneously recruiting and activating different immune effector cells to the tumor site result in potent tumor-cell elimination by the above different immunologic killing mechanisms

The interaction of T cells and tumor cells, which is mediated by BsAb, initiates the killing process of T cells, including activation of CD3, formation of immunologic synapses, activation and proliferation of T cell, secretion of cytokines and cytotoxic granules, and lysis of tumor cells [23]. The activated CD8^+^ and CD4^+^ T cells lyse cancer cells predominantly through perforin and granzyme B. The activated T cells secrete various cytokines such as IFN-γ, TNF, IL-2, IL-6, and IL-10 [24]. In addition to the above mechanism, triomabs can recruit other immune cells such as NK cells, macrophages which could kill tumor cells and mediate the co-stimulation between T cells and accessory cells [20]. Simultaneously recruiting and activating different immune effector cells to the tumor site result in potent tumor-cell elimination by the different immunologic killing mechanisms mentioned above [25] (Fig. 2).

At present, although the clinical outcome of BsAB is less satisfied in solid tumors than in hematologic malignancies [26, 27], there are abundant ongoing studies, and some products have entered clinical trials. In this review, we will mainly summarize the correlated studies of BsAb in solid tumors (Table 1) and discuss the challenges and corresponding approaches in clinical application. This review will focus on commonly expressed antigens on solid tumors, such as EpCAM, HER family, CEA, and PSMA, and BsAB targeting these antigens are also extensively investigated and have demonstrated a great potential in cancer immunotherapy.

**Table 1:** Clinical trials of EpCAM, HER family, CEA- and PSMA-related BsAbs in solid tumors

BsAb	Targets	Format	Function	Diseases	Phase	Stage	Identifier
Catumaxomab	EpCAM/CD3	Triomab	Redirect T cells and accessory cells	Ovarian cancer	II	Completed	NCT00189345
Catumaxomab	EpCAM/CD3	Triomab	Redirect T cells and accessory cells	EpCAM positive tumor, malignant ascites	II/III	Completed	NCT00836654
Catumaxomab	EpCAM/CD3	Triomab	Redirect T cells and accessory cells	Gastric cancer, adenocarcinoma	II	Completed	NCT00464893
Catumaxomab	EpCAM/CD3	Triomab	Redirect T cells and accessory cells	Gastric adenocarcinomas	II	Active, not recruiting	NCT01504256
Catumaxomab	EpCAM/CD3	Triomab	Redirect T cells and accessory cells	Ovarian cancer	II	Completed	NCT01246440
Catumaxomab	EpCAM/CD3	Triomab	Redirect T cells and accessory cells	Gastric peritoneal carcinomatosis	II	Terminated	NCT01784900
Catumaxomab	EpCAM/CD3	Triomab	Redirect T cells and accessory cells	Epithelial cancer	I	Terminated	NCT01320020
Catumaxomab	EpCAM/CD3	Triomab	Redirect T cells and accessory cells	Malignant ascites	III	Completed	NCT00822809
MT110	EpCAM/CD3	BiTE	T cell recruitment	EpCAM-positive solid tumors	I	Completed	NCT00635596
Ertumaxomab	HER2/CD3	Triomab	Redirect T cells and accessory cells	Breast cancer	II	Terminated	NCT00452140
Ertumaxomab	HER2/CD3	Triomab	Redirect T cells and accessory cells	Breast cancer	II	Terminated	NCT00522457
Ertumaxomab	HER2/CD3	Triomab	Redirect T cells and accessory cells	Her2 positive advanced solid tumors	I/II	Terminated	NCT01569412
HER2Bi-aATC	HER2/CD3	T cells armed with BsAbs	Activated T cells	Her2-positive neoplasms	I	Recruiting	NCT02662348
EGFRBi-aATC	EGFR/CD3	T cells armed with BsAbs	Activated T cells	Advanced solid tumors	I	Terminated	NCT01081808
MDX-447	EGFR/CD64	2(Fab’)	Active monocytes	Brain and central nervous system tumors	I	Completed	NCT00005813
MM-111	HER2/HER3	HSA body	Blockade of 2 receptors	HER2 amplified solid tumors	I	Completed	NCT00911898
MM-111 + Herceptin	HER2/HER3	HSA body	Blockade of 2 receptors	Breast cancer	I	Completed	NCT01097460
MCLA-128	HER2/HER3	Full Length IgG1	Blockade of 2 receptors	Malignant solid tumors	I/II	Recruiting	NCT02912949
MM-141	HER3/IGF-IR	scFv-IgG	Blockade of 2 receptors	Hepatocellular carcinoma	I	Completed	NCT01733004
MM-141	HER3/IGF-IR	scFv-IgG	Blockade of 2 receptors	Pancreatic cancer	II	Recruiting	NCT02399137
LY3164530	EGFR/MET	OrthoFab-IgG	Blockade of 2 receptors	Advanced or metastatic cancer	I	Active, not recruiting	NCT02221882
TargomiRs	EGFR/EDV	Unclear	Delivery of nanoparticles	MPM and NSCLC	I	Recruiting	NCT02369198
EGFR(V)-EDV-Dox	EGFR/EDV	Unclear	Delivery of nanoparticles	Glioblastoma	I	Recruiting	NCT02766699
AMG211	CEA/CD3	BiTE	T cell recruitment	Gastrointestinal cancer	I	Recruiting	NCT02760199
AMG211	CEA/CD3	BiTE	T cell recruitment	Gastrointestinal cancer	I	Recruiting	NCT02291614
AMG211	CEA/CD3	BiTE	T cell recruitment	Gastrointestinal adenocarcinomas	I	Recruiting	NCT01284231
RO6958688	CEA/CD3	IgG-based	T cell recruitment	CEA-positive solid tumors	I	Recruiting	NCT02324257
RO6958688+Atezolizumab	CEA/CD3	IgG-based	T cell recruitment	CEA-positive solid tumors	I	Recruiting	NCT02650713
RO6895882	CEA/IL2	ScFv-IgG	The delivery of cytokines	CEA-positive solid tumors	I	Completed	NCT02004106
TF2	CEA/HSG	Dock and lock	Radioimmunotherapy	Colorectal cancer	I	Completed	NCT00860860
TF2	CEA/ HSG	Dock and lock	Radioimmunotherapy	Small cell lung cancer	I/II	Completed	NCT01221675
TF2	CEA/ HSG	Dock and lock	Immuno-PET	Medullary thyroid carcinoma	I/II	Completed	NCT01730638
TF2	CEA/ HSG	Dock and lock	Immuno-PET	Breast carcinoma expressing CEA	I/II	Ongoing	NCT01730612
TF2	CEA/ HSG	Dock and lock	Radioimmunotherapy	Colorectal cancer	I	Terminated	NCT01273402
TF2	CEA/ HSG	Dock and lock	Immuno-PET	Colorectal cancer	II	Completed	NCT02587247
Anti-CEAxanti-DTPA	CEA/di-DTPA-131	scFv-IgG	Radioimmunotherapy	Medullary thyroid carcinoma	II	Completed	NCT00467506
BAY2010112	PSMA/CD3	BiTE	T cell recruitment	CRPC	I	ongoing	NCT01723475
MOR209/ES414	PSMA/CD3	ScFv-Fc-scFv	T cell recruitment	mCRPC	I	Recruiting	NCT02262910

The details of Table 1 derived from http://clinicaltrials.gov/

## Targeting antigens

### EpCAM

EpCAM (CD326, 17-1A) is a 39–40 KDa transmembrane glycoprotein that functions as adhesion molecule [28, 29]. EpCAM is expressed by majority of normal epithelial tissues including lung, colon, pancreas, bile ducts, breast, as well as embryonic stem cells [30, 31]. Similar to CD44, CD133, and CD166, EpCAM is also considered as a cancer stem cell (CSC) marker [32–34]. The expression of EpCAM is correlated with epithelial cell proliferation, differentiation, and migration [35–37]. EpICD, as the intracellular domain of EpCAM, is associated with the Wnt pathway which regulates gene transcription when translocated into the nucleus resulting in cell proliferation and tumor formation [32, 38]. EpCAM is expressed on certain carcinomas including ovarian cancer, breast cancer, lung cancer, pancreas cancer, colorectal cancer, head and neck squamous-cell carcinoma (HNSCC), and gastric cancer [39, 40]. Overexpression of EpCAM was detected in 35.6% breast cancer samples by the immunohistochemical method and was related to poor prognosis [41]. A study demonstrated that high expression of EpCAM was a poor prognostic indicator of breast cancer with node-positive [42]. Similarly, a retrospective study found that EpCAM was overexpressed in 68.8% epithelial ovarian cancer (EOC) and was associated with reduced survival time, especially in stage III–IV and poorly differentiated subtype [43]. EpCAM was highly expressed in 86.5% of non-small cell lung cancer (NSCLC) patients [39]. Moreover, high level of EpCAM expression was detected in more than 90% of HNSCC patients [35]. Therefore, bispecific antibody targeting EpCAM provides an attractive choice for immunotherapy of those cancers. Triomab (e.g., catumaxomab) and BiTE (e.g., MT110) are two major types of anti-EpCAM x anti-CD3 BiAbs.

Catumaxomab (Removab) is an intact trifunctional bispecific antibody consisting of a murine IgG2a targeting EpCAM, a rat IgG2b targeting CD3, and Fc fragment recruiting different immune effector cells [44]. It was approved by the European Union for the treatment of malignant ascites in April 2009 [45]. The trifunction of catumaxomab was assessed by co-culture of tumor spheroids of FaDu cell line (HNSCC) with peripheral blood mononuclear cells (PBMCs). The results indicated that three functional parts of catumaxomab were essential for the entire antitumor activity [46]. Schmitt et al. investigated the opsonization mediated by catumaxomab with co-culture of tumor cells and PBMCs. In their studies, opsonization with catumaxomab caused the activation of PBMCs and destroyed EpCAM-positive tumor cells [44]. To evaluate the immunomodulatory effects of catumaxomab, Zitvogel et al. developed in vitro experimental model of malignant ascites system. They found that catumaxomab activated and transformed T cells to inflammatory CD4^+^ and CD8^+^ Th1 cells, and it stimulated the secretion of IFN-γ. In addition, catumaxomab promoted CD16^+^ cells to express TRAIL and costimulatory molecules CD40 and CD80 [22]. Another group investigated the immunological changes in six patients with malignant ascites after intraperitoneal administration of catumaxomab. They found the accumulation of NK cells, macrophages, and T cells in the peritoneal cavity. At the same time, CD69 and CD38, the activation molecular of T cells, were induced by the intraperitoneal (i.p.) catumaxomab infusion. Catumaxomab promoted the secretion of IFN-γ and IL-2, whereas its functions were inhibited in the immunosuppression microenvironment of ascites in vitro [47]. In an open-label, dose-escalation clinical trial, 16 patients with EpCAM-positive solid tumors were enrolled and treated with catumaxomab. The antitumor efficacy was not optimal: two patients had stable disease, nine patients had disease progression, and the remaining patients were not evaluable. The study results determined that the maximum tolerated dose (MTD) of intravenous catumaxomab was 7 μg/kg. The cytokine release-related symptoms and hepatotoxicity were considered as the major adverse events (AEs) [48]. In a large phase II/III trial, 258 patients with malignant ascites due to epithelial cancer were enrolled. The total patients were comprised of 129 ovarian cancer patients and 129 non-ovarian cancer patients. In each group, 85 patients were treated with paracentesis plus catumaxomab, and the other 44 patients were treated with paracentesis alone. The difference of the time to next paracentesis in the catumaxomab group (77 days) and in the control group (13 days) was significant. The puncture-free survival and overall survival (OS) also exhibited beneficial trend in the catumaxomab group. In addition, compared with the control group, fewer signs and symptoms of ascites were observed in catumaxomab-treated patients. The side effects related to catumaxomab were reversible and manageable [49]. In another phase I clinical trial, a total of 21 patients with NSCLC were recruited, and 15 of them were treated with catumaxomab and were evaluated for the dose limiting toxicity (DLT) and MTD. The five dose-escalation levels ranged from 2 μg/kg to 7.5 μg/kg. In dose level IV and V, grade 3 and 4 elevations of ALT, AST, and γ-GT were observed, which were identified as the DLT. Nevertheless, the elevation of liver enzymes was reversible. The MTD was determined in dose level III (5 μg of catumaxomab). Moreover, it did not observe the HAMA /HARA (human anti-mouse/human anti-rat antibody) within 28 days in 15 evaluable patients [50]. Twenty-three patients with malignant ascites due to refractory ovarian cancer were treated with i.p. catumaxomab in phase I/II study. The i.p. of catumaxomab significantly decreased the ascites production. During the infusion, just 1 of 23 patients required a paracentesis. Promisingly, severe adverse events were not observed [51].

MT110 (solitomab) is a BiTE bispecific antibody consisting of two scFvs. One of the scFvs binds to EpCAM expressed on tumor cells, and the other binds to CD3 on T cells [23]. BsAb which targets EpCAM and CD3 prolonged the contact time between lymphocytes and cancer cells [52]. Via MT110, T cells could potently recognize and lyse target tumor cells. The mechanism of lysis predominantly depends on the pore forming and apoptosis. The lysis process including caspase activation, PARP cleavage, and DNA fragmentation was mainly mediated by granzyme B and perforin [23]. MT110 showed potent antitumor effect against chemotherapy-resistant ovarian cancer cell lines. When incubated with autologous tumor-associated T cells and EpCAM^+^ ovarian cancer cells derived from ascites, MT110 upregulated the expression of T cell activation markers and enhanced its cytoxicity to malignant cells [53]. CSCs might be responsible, at least partly, for the resistance to chemotherapy and recurrence of hepatocellular carcinoma (HCC) [54, 55]. EpCAM was considered as a CSC marker in HCC [56]. On the basis above, Blaudszum et al. generated an EpCAM/CD3 BiTE using the scFvs of anti-EpCAM monoclonal antibody 1H8 and anti-CD3 monoclonal antibody. Their results indicated that 1H8/CD3 effectively eradicated CD133^+^ EpCAM^+^ HCC CSCs and EpCAM^+^ HCC cells in vitro and in vivo [57]. EpCAM/CD3-BiTE potently killed the colon cancer cell line at a low effector-to-target ratio in vitro and significantly restricted the ovarian cancer growth in a xenograft model [58]. Another study showed that MT110 eliminated colorectal cancer cells and stem cells [59]. Some studies also demonstrated that MT110 could eradicate the primary cancer cells and the CSCs of pancreatic cancer in vivo and in vitro [60, 61].

Mus110 is a BiTE bispecific antibody to murine EpCAM and murine CD3, and its structure is similar to MT110. In breast cancer and lung cancer mouse models, Mus110 showed potent antitumor activity as low as 5 μg/kg, but mice could tolerate a high dose of mus110 up to 400 μg/kg [31]. Studies have shown that adverse events of mus110 in mice were mainly due to an acute T cell activation. The therapeutic window and target-related side effects of mus110 in mice might be a prediction for MT110 in human [62, 63]. As we all know, compared with triomabs, BiTEs are not able to mediate ADCC, CDC, and ADCP for lacking Fc region. However, the BiTE antibody against EpCAM and CD16 recruited innate immune cells and then induced effective ADCC, as well as enhanced the killing of human carcinoma overexpressing EpCAM [64]. IL-2-activated lymphocytes armed with trifunctional BsAb against EpCAM and CD3 induced long-lasting antitumor effects in melanoma mice model. Encouragingly, the graft-vs-host disease (GVHD) was not observed [65]. A study demonstrated that lymphocytes overexpressing TRAIL in combination with EpCAM × CD3 bispecific antibody prolonged the exposure time of TRAIL with its receptors on tumor cells and enhanced the antitumor response [66]. Besides, a novel recombinant antibody E3Bi enhanced the specific cytotoxicity of activated T cell (ATC) in tumor cell lines with high EpCAM expression and significantly inhibited tumor growth in mice model [67]. A BsAb HEA125 × OKT3 co-targeting EpCAM on EpCAM^+^ tumor cells and CD3 on T cells mediated the interaction of tumor cells and T cells which resulted in the formation of an immune synapse and activation of T cell [68].

### HER family

The receptor tyrosine kinase family known as the HER family consists of four members: EGFR (also known as ErbB1/HER1), HER2, HER3, and HER4. HER1–4 play a pivotal role in controlling and regulating cell growth, differentiation, migration, and death [69, 70]. EGFR as a tumor-associated antigen overexpressed on the cell surface of various malignant tumors, such as NSCLC, glioblastoma, pancreatic cancer, HNSCC, renal cancer, and colorectal cancer (CRC) [71]. HER3 has been identified as a critical molecule in the interaction with ligand as well as PI3K signaling pathway [72]. Compared with other members, HER4 is less known to us. Study demonstrated that HER4 is a favorable prognostic marker for OS in patients with breast cancer [73]. Based on the above reasons, the HER family members are as attractive targets for immunotherapy, especially in the application of BsAb.

The application of anti-EGFR monoclonal antibody (such as cetuximab and panitumumab) in EGFR overexpressing tumors has been marketed for many years [74]. However, there are studies demonstrated that the therapeutic outcome of anti-EGFR mAbs is not well satisfying in patients with *KRAS* and *BRAF* genes mutated CRC [75]. T cell engaged BiTE antibodies using the binding domains of cetuximab, and panitumumab remained potent antitumor activity in *KRAS* and *BRAF* mutation of CRC cell lines and in xenograft models [76]. Glioblastoma overexpressed wild type EGFR, EGFRvIII, and HER2, so they were all considered as attractive immunotherapy targets [77]. BsAb that target EGFR and HER2 may be an effective strategy for the treatment of glioblastoma. A group examined the antitumor activity of the ATC armed with chemically heteroconjugated anti-CD3 × anti-HER2 (HER2Bi) and/or anti-CD3 × anti-EGFR (EGFRBi). It was demonstrated that the armed ATC significantly killed malignant glioma lines (U87MG, U118MG, and U251MG) and primary glioblastoma lines. Moreover, the increased secretion of three Th1 cytokines (IFN-γ, GM-CSF, and TNF-α) and one Th2 cytokine (IL-13) had been detected [78]. EGFRBi-armed CIK cells showed significant antitumor effects in EGFR-positive glioblastoma in vitro and in vivo [79]. A clinical study led by Solomon et al. examined the safety of EGFR-targeted, paclitaxel-loaded minicells (^EGFR^minicells_Pac_). Among 22 patients that completed cycle 1 treatment, ten patients achieved stable disease, and 12 had progressive disease. The most common treatment-related AEs were chills and pyrexia. The number of 1 × 10^10^ CIK cells was considered to be the MTD. In general, the study reported that the ^EGFR^minicells_Pac_ could be safely administered to patients with advanced solid tumors [80].

Evidences showed that fully human HER2/CD3 BsAb potently delayed the growth of breast cancer by stimulating the activation and proliferation of tumor-infiltrating lymphocytes [81]. More recently, a phase I trial was conducted by Lum and colleagues. Eight castrate resistant prostate cancer (CRPC) patients were treated with HER2Bi-armed ATC at infusion 2.5, 5, 10, and 20 billion units. One patient achieved partial response, and three of seven patients had a remarkable decline in their PSA levels. The Th1 cytokines of two patients had increased. In addition, no dose limiting toxicities were observed [82]. Another phase I clinical trial was conducted to test the safety and efficacy of HER2Bi-armed ATC in combination with interleukin 2 (IL-2) and granulocyte-macrophage colony stimulating factor (GM-CSF) in 23 patients with stage IV breast cancer. Thirteen of 22 evaluable patients achieved a stable disease condition, and the remaining patients had progressive disease. The median OS for all patients was 36.2 months, 57.4 months for the HER2 3^+^ group, and 27.4 months for the HER2 0-2^+^ group. The major side effects including chills, fever, headache, fatigue, and hypotension, were controllable and reversible. The MTD was not reached. Encouragingly, this strategy induced endogenous cytotoxicity and cytokine responses in evaluable patients [83]. Ertumaxomab, as a trifunctional antibody, could eliminate tumor cell lines regardless of HER2 expression level. However, trastuzumab-mediated cytotoxicity depends on the high expression of HER2 since the HER2 binding sites for trastuzumab and ertumaxomab are located in different positions [84]. A phase I clinical trial was conducted by Kiewe et al. to determine the safety and efficacy of ertumaxomab in patients with metastatic breast cancer. Fifteen of 17 enrolled patients completed the study with three ascending doses of ertumaxomab (10–200 Ag). There were 5 out of 15 evaluable patients showed antitumor response including one with complete response, two with partial responses, and two with stable disease. The patients infused with 150 μg/kg and 200 μg/kg developed severe toxicities. Therefore, 100 μg/kg is suggested as the MTD. Human anti-mouse antibody (HAMA) was induced in 4 out of 16 evaluable patients (25%) on day 41 [85]. Fourteen patients with HER2-positive advanced solid tumors were enrolled in another phase I trial. Patients were treated with the trifunctional antibody ertumaxomab in a weekly escalating dosing regimen. The clinical response to ertumaxomab treatment was seen in 3 out of 11 evaluable patients, including one partial remission and two disease stabilizations. The treatment-related toxicities were mild and completely reversible [86].

The tetramerized bispecific antibody targeted EGFR and CD16 simultaneously and then exhibited cytotoxicity against EGFR-expressing tumor cells [87]. MDX-447 is a bispecific antibody that combined humanized Fab anti-FcγRI (CD64) and humanized Fab anti-EGFR [88]. Another bispecific antibody MDX-210 co-targeting HER2/neu and FcγRI increased the efficacy in vitro when combined with granulocyte-colony stimulating factor (G-CSF) in breast cancer patients overexpressing HER2/neu [89]. Therefore, a phase I clinical study was conducted to determine the safety and efficacy of MDX-447 with and without recombinant human G-CSF in patients with advanced solid tumors. The study results indicated that MDX-447 alone was generally well tolerated, but the combination of MDX-447 and G-CSF was not [90].

Targeting individual members of HER family such as EGFR or HER2 led to limited antitumor activity. The BsAb not only redirected effector cells to the target tumor cells but also bound to two receptors and blocked the downstream signaling pathway. Anti-EGFR/HER2 bispecific antibody effectively suppressed the growth of breast tumor [8]. Another BsAb targeting HER2/HER3 overcomes the heregulin-induced resistance to PI3K inhibition in prostate cancer [91]. MM-111, a bispecific antibody consisting of human anti-HER2 and anti-HER3 scFv linked by modified human serum albumin (HSA) blocked HER3 and PI3K pathway in the HER2-overexpressing cells and inhibited tumor growth in xenograft models. MM-111 combining with trastuzumab or lapatinib showed potent antitumor ability in the HER2-overexpressing tumors [92]. The phosphorylation of EGFR and HER3 activated the downstream Ras/MAPK and (PI3K)/AKT signaling pathways which contributed to the cell growth and proliferation. The monospecific antibodies of EGFR or HER3 cannot completely inhibit the proliferation and survival signals [93, 94]. Sliwkowski et al. constructed a two-in-one antibody against HER3 and EGFR (namely MEHD7945A) and tested the function in vitro and in vivo. The study results showed that MEHD7945A not only potently inhibited receptor phosphorylation of EGFR and HER3 but also enhanced gemcitabine-mediated cytotoxicity in vitro and in vivo. Besides, the dermatologic toxicity of MEHD7945A was significantly less than monospecific antibody in xenograft models [95]. In addition, the binding of HER family members and other receptors also enhanced the therapeutic outcome. For instance, EGFR × c-MET bispecific antibody JNJ-61186372 enhanced the killing of EGFR mutant lung cancer cells [9]. BsAb co-targeting EGFR and VEGFR2 promoted the antitumor activity by inhibiting phosphorylation of the receptors and blockade PI3K/AKT and MAPK signaling pathways [96]. EGFR and the insulin-like growth factor-1 receptor (IGF-1R) play an essential role in cell proliferation and tumor progression. Therefore, the bispecific antibody XGER targeting EGFR and IGF-1R exhibited potent antitumor efficacy [97]. Negrin et al. investigated the ability of BsAb anti-HER2 × cancer antigen-125 (CA125) with CIK cells against primary ovarian carcinomas. The results suggested that the cytolytic activity of CIK cells with BsAb was significantly higher than CIK cells alone [98]. Study showed that in primary breast cancer, 65% were positive for CEA, 19% were positive for HER2, and 12% expressed both antigens. Therefore, the bispecific antibody simultaneously targeting HER2 and CEA on the same cell obviously enhanced tumor localization [99].

### CEA

CEA is a 180–200 KDa glycoprotein that belongs to the CEA-related cell adhesion (CEACAM) superfamily [100, 101]. CEA is expressed at low levels in various normal tissues including colon, stomach, esophagus, tongue, cervix, and prostate [102]. Under physiological conditions, CEA is expressed on the apical surface and luminal portion of normal epithelial cells [101]. But in cancer tissues, CEA is overexpressed and lose the polarized distribution. Besides, CEA was cleaved from the surface of cancer cells by phospholipase, which resulted in the increase of serum CEA [103]. Blood levels of CEA are currently used as a diagnostic and prognostic marker, as well as a monitoring index in patients after treatment [104, 105]. It was demonstrated that serum levels of soluble CEA did not affect the tumor suppression by CEA/CD3 BsAbs [106, 107]. CEA-overexpressing malignant cancers included colorectal, gastric, lung, breast, pancreatic, and other cancers [108]. CEA, as a well characterized tumor-associated antigen (TAA), plays a vital role in cancer adhesion, migration, and invasion [109]. Thus, it has become a pivotal target for the immunotherapy, including antibody-based treatments of CEA-positive solid tumors [110].

MEDI-565 (AMG211, MT111), as a BiTE antibody mediating T cell-directed cytotoxicity towards CEA positive tumor cells, was positively correlated with CEA antigen density regardless of the mutational status of the tumor cell lines, including BRAF, KRAS, PTEN, PI3KCA, and TP53 [111]. MEDI-565 recognized a nonlinear epitope in the full-length but not a short splice variant of CEA. The CEA splice variant neither affected the binding of MEDI-565 and full-length CEA nor inhibited MEDI-565-induced T-cell activation and cytotoxicity [112]. In vitro, normal donor- and cancer patient-derived T cells redirected by MEDI-565 induced cytotoxicity to CEA-positive tumor cells which were derived from patients with metastatic colorectal cancer and previously treated with chemotherapy. In mice xenografted model, the MEDI-565 also significantly inhibited tumor growth at a low concentration (1 ng/ml) without the assistance of costimulatory agents [106]. In a multicenter phase I, open-label study (NCT01284231), a total of 39 patients with advanced gastrointestinal adenocarcinomas were enrolled and were intravenously injected with MEDI-565 over 3 h on days 1 through 5 in 28-day cycles with dexamethasone premedication. The study results demonstrated that 11 patients had stable disease. The median overall survival for 39 patients was 5.5 months, and the MTD of MEDI-565 was 5 mg. Nausea, vomiting, abdominal, and fatigue were considered as the most common adverse effects. During the treatment, high-level antidrug antibodies were detected in 19 patients. Like other BiTEs, MEDI-565 showed rapid clearance and a short half-life [113]. As we know, recent researches have focused on checkpoint blockade. MEDI-565 in combination with anti-PD1 and/or anti-PD-L1 antibodies could significantly enhanced T cell cytotoxicity activity [114]. Bacac et al. developed the CEA TCB (RO6958688) that is a novel IgG-based T-cell bispecific (TCB) antibody. CEA TCB is a head-to-tail 2:1 T cell bispecific antibody that harbors bivalent binding site for CEA and monovalent binding site for CD3 [115]. CEA TCB significantly eliminated the CEA-expressing xenograft tumor in mice models. Moreover, number of immune cells infiltrating in the tumor tissues were observed, which resulted in a highly inflamed tumor microenvironment. The group also demonstrated that the activity of CEA TCB positively related to CEA expression. The efficient target cell lysis required at least approximately 10,000 CEA-binding sites [116]. Another study results suggested that CEA TCB potently increased the number of tumor-associated T cells and induced death of tumor cell within 24 h in vitro and in vivo. In addition, the investigators also observed the prolonged interactions between multiple T cells and tumor cells in vivo by fluorescence imaging [117]. A phase I study (NCT02324257) led by Hoffmann-La Roche which focused on the safety and feasibility of CEA TCB to patients with advanced CEA-positive solid tumors is currently ongoing.

A novel bispecific antibody BiSS (Bispecific antibody with Single domain, Single domain antibodies) was constructed by tandemly linking two single domain antibodies, anti-CEA, and anti-CD16. BiSS exhibited potent recruitment of NK cells and cytotoxicity to CEA-positive tumor cells, HT29 and LS174T. In an in vivo study, BiSS also significantly limited cancer progression [118]. Another single domain antibody-based bispecific antibody (anti-CD16 × anti-CEA) ss-Fc fused with CH3 “knobs into holes” also showed the potent antitumor activity in vitro and in vivo [119]. Compared with single-chain tandem scFvs (e.g., BiTE), two-chain diabodies induced T cell-activated proliferation in a target cell-dependent manner, which could reduce the toxicity to normal tissues [120]. However, unbalanced expression of the two chains of diabody is a limitation for the function of it. The incorporation of a 2A self-processing peptide derived from foot-and-mouth disease virus and a two-chain diabody gene balanced the secretion of diabody chains and maximized the final amount of assembled diabody [121]. Due to the important role in the cell cycle, tumor necrosis factor alpha (TNF-α) is considered as a potential tumor treatment strategy. However, the concentration of TNF-α is not high enough to exhibit its antitumor activity. Therefore, Azria et al. developed a bispecific antibody anti-TNF-α × anti-CEA in combination with TNF-α and radiotherapy to enhance the tumor growth control of pancreatic tumor xenografts in nude mice [122].

**Fig. 3:**
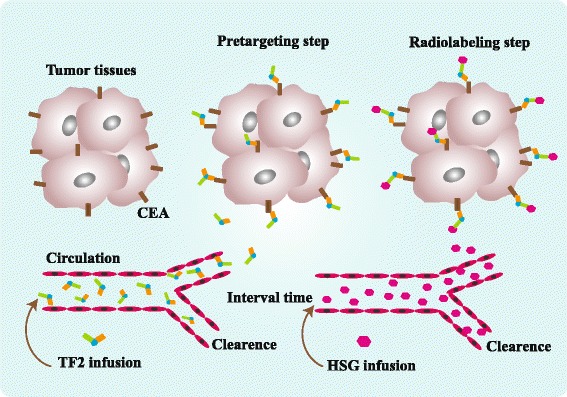
The classical two-step protocol of pre-targeted radioimmunotherapy (taking TF2 as an example). The first step is to administer the TF2 to blood and offer adequate time for tumor uptake and clearance of excess TF2 in circulation. The second step is to infuse HSG. Finally, excess HSG is cleared from the bloodstream by kidneys

Radioimmunotherapy (RIT) is a molecular targeted therapy that uses mAb targeting specific tumor antigens to deliver radionuclides to tumor sites and kill tumor cells [123]. The application of RIT in treatment of hematologic malignancies achieved a gratifying outcome, but not in solid tumor [124]. The main restriction of RIT in solid tumor is bone marrow exposure and dose-limiting hematological toxicity caused by the moderate tumor/non-tumor ratios [125]. With the development of recombinant and humanized mAbs, pretargeting radioimmunotherapy (pRIT) using BsAb is becoming a potential therapeutic approach [126]. The classical two-step protocol of pRIT is to administer the BsAb to blood with adequate time for tumor uptake and clearance of excess BsAb in circulation, and then infuse the radiolabeled hapten, finally excess radiolabeled hapten is cleared from the bloodstream [127] (Fig. 3). Due to the extensive expression profile of CEA in several cancers, pretargeting BsAbs anti-CEA × anti-hapten were studied by numerous investigators.

TF2 is a trivalent humanized bispecific antibody that composed of two anti-CEA Fab fragments and an anti-histamine-succinyl-glycine (HSG) Fab fragment linked by the dock and lock method [128, 129]. The pre-targeting strategy using bispecific anti-CEA × anti-diethylenetriaminepentaaceticacid (DTPA) antibody and radiolabeled peptide significantly improved the tumor/blood ratios due to the rapid clearance of the radiolabeled peptide from the circulation [130]. The anti-CEA/anti-DTPA-indium complex BsAb combined with radiolabeled liposomes to carry high radionuclide activities and thus optimized the pre-targeting RIT of solid tumors [123]. The anti-CEA/anti-1, 4, 7, 10-tetraazacyclododecane-1, 4, 7, 10-tetraacetic acid (DOTA) bispecific antibody exhibited low tumor targeting and rapid blood clearance in a xenograft mouse model, but the specific tumor uptake and low normal tissue accumulation of the BsAb still improved the efficacy of RIT [131]. Karacay et al. made comparison between pretargeted peptide and directly radiolabeled IgG in a human colon cancer xenograft. The study demonstrated that the BsAb pre-targeting strategy sufficiently increased the dose of radioactivity in tumors and caused less hematologic toxicity than conventional RIT [132]. In order to increase tumor-to-blood ratios, the three-step protocol namely adding an avidin chase procedure on the basis of two-step was developed. The chase rapidly cleared the BsAb level from circulation, thereby reduced hapten concentration in blood and bone marrow exposure [133]. Thirteen patients with non-medullary thyroid carcinoma (non-MTC) and nine patients with medullary thyroid carcinoma (MTC) were enrolled in a phase I optimization clinical trial. The 75 mg/m^2^ dose of BsAb was infused to 11 patients. 40 mg/m^2^ dose of BsAb was infused to the remaining patients. Five days later, all patients received 1.9–5.5 GBq of 131I-di-DTPA. The results suggested that 40 mg/m^2^ of BsAb and 5-day interval could be a better schedule for tolerable toxicity [134]. A phase II clinical trial (NCT00467506) was conducted to determine the efficacy and safety of anti-CEA × anti-DTPA BsAb and 131I-di-DTPA-indium bivalent hapten in patients with progressive metastatic MTC. Forty-five patients enrolled the study, but 42 completed the designed procedure and were evaluable for efficacy, adverse events, and response assessment. The study results indicated potent therapeutic responses including disease control in 76% of patients. Median progression-free survival (PFS) was 13.6 months, and median OS was 43.9 months. The main subacute adverse event was bone marrow exposure-related hematologic toxicity. Moreover, a significant increase of calcitonin doubling time (DT) in 56.7% of patients after pRIT was observed. The calcitonin DT was seen as an independent prognostic factor [135]. In another phase I/II trial (NCT01221675) by Centre René Gauducheau, nine patients with CEA-expressing advanced lung cancer were treated with TF2 and the IMP288 bivalent HSG peptide. The procedure included a pre-therapeutic imaging session and a therapy session. The pre-targeting delay was 24 or 48 h. In the end, one patient died (not considered treatment related) and eight patients were evaluated for pharmacokinetics, dosimetry, toxicity, and response. The study results suggested that increased the TF2 dose and shortened the pre-targeting delay were benefit for tumor uptake without increase of the toxicity to normal tissues. All patients were pretreated with an antihistamine and corticosteroid before each TF2 and peptide infusion thus only one patient was detected with human anti-human antibody (HAHA) against TF2 > 50 ng/ml [136].

The BsAb with radiolabeled hapten is a sensitive diagnostic tool. Bispecific antibody pre-targeting positron-emission tomography (PET) with a ^68^Ga- or ^18^F-hapten-peptide showed specific targeting in CEA-positive tumor and low ingestion in normal tissues and CEA-negative tumors [137]. BsAb anti-CEA × anti-hapten with an ^124^I-labeled hapten-peptide significantly increased tumor uptake and tumor-to-blood ratios in comparison to directly radiolabeled antibodies. Furthermore, the BsAb pre-targeting showed rapid clearance from normal tissues and clear visualization of tumor within 1–2 h [138]. TF2 pre-targeting CEA on the surface of tumor cells followed by the addition of Ga-labeled hapten could be obviously sensitive in the visualization of CEA [139]. Besides, it was proved that BsAb pre-targeting was highly selective for imaging micrometastatic tumor and showed better contrast ratio than ^18^F-FDG. Hence, single photon emission computed tomography (SPECT) and PET pre-targeted with TF2 could be a promising approach to improve imaging of metastatic CEA-positive malignancies [140]. Compared with conventional ^99^mTc-labeled CEA-specific F(ab’), the BsAb-pretargeted ^99^mTc radiotracer increased 10-fold of the radioactivity signal and showed faster clearance from the circulation and other normal tissues [141]. Fourteen patients with primary colorectal cancer were included and administered with anti-CEA × anti-Di-DTPA BsAb and a ^111^In-labeled di-DTPA peptide to assess the imaging effect. One of three patients that received ^111^In-peptide alone showed low tumor uptake. In 9 of 11 patients that received BsAb in combination with ^111^In-peptide, tumors were observed. The results suggested that pretargeting imaging was a promising diagnostic strategy using low dose BsAb and ^111^In-labeled peptide, with an optimal delay of 4 days between infusions of the two agents [142]. Another clinical trial on immune-PET using anti-CEA and 68Ga-labeled peptide in patients with metastatic medullary thyroid carcinoma showed that 30 h was the most favorable delay [143].

### PSMA

PSMA is a membrane bound protein that is selectively expressed on the surface of prostate cancer cells as well as in the neovasculature of most solid tumors [144, 145]. PSMA is expressed across all stages of prostate cancer, and the expression level is inversely correlated with androgen levels [145]. PSMA plays an essential role in the progression of prostate cancer through MAPK-ERK1/2 and PI3K-AKT pathway [146]; besides, it can be used as the target of imaging agent to detect the metastatic tumor sites [147]. Therefore, PSMA is considered as an attractive target for the immunotherapy of prostate cancers.

BAY2010112 (AMG212, MT112), as a PSMA/CD3-bispecific BiTE antibody, bound to PSMA which was expressed in prostate cancer cell lines and PSMA cDNA transfected cell lines, and mediated T cells to eliminate target cells in vitro. The BiTE antibody potently suppressed tumor growth at a dose of 0.005 mg/kg daily intravenous (i.v.) administration [144]. BYA2010112 induced target-dependent activation and cytokine released by T cells. T cells exhibited potent cytotoxicity against PSMA-positive cell lines with the help of BAY2010112 in vitro. Compared to i.v. administration, subcutaneous (s.c.) injection of BAY2010112 significantly inhibited tumor formation and induced tumor regression in the subcutaneous xenograft immunodeficient NOD/SCID mice. In addition, the bioavailability of BAY2010112 was approximately 18% after s.c. administration in mice [148]. At present, a dose-escalation phase I clinical trial (NCT01723475) on BAY2010112 is ongoing. Patients with castration resistant prostate cancer will be recruited and treated with different dosages of BAY2010112. The primary objectives of this study are to determine the safety, tolerability, and MTD of BAY2010112. The secondary objectives are to assess the pharmacokinetics and the clinical efficacy of BAY2010112. MOR209/ES414 is a novel humanized BsAb which is designed to treat metastatic castration-resistant prostate cancer (mCRPC) by redirecting T cell cytotoxicity against prostate cancer cells expressing PSMA. MOR209/ES414 induced T cell activation and proliferation, and lysed tumor cells in vitro. In murine xenograft models, MOR209/ES414 also showed significant inhibitory effect on tumor and prolonged the survival time. The half-life period of MOR209/ES414 was 4 days in the peripheral blood of NOD/SCIDγ (NSG) mice [149]. A phase I study (NCT02262910) of MOR209/ES414 in patients with mCRPC is ongoing. The study is conducted by Aptevo Therapeutics to evaluate the tolerability, pharmacokinetics (PK), pharmacodynamics (PD), immunogenicity, cytokine response, and clinical activity of MOR209/ES414. Anti-PSMA × anti-CD3 BsAb could specifically bind to CD3-expressing Jurkat cells and PSMA-expressing C4-2 cells, as well as efficiently promoted the function of T cells to lyse target cells. PSMA × CD3 diabody showed efficient inhibition of tumor growth in C4-2 tumor xenografts [150, 151]. After activation, CD4^+^ and CD8^+^ T cells expanded and killed prostate cancer cells mainly through the perforin-granzyme-based pathway, while the FasL pathway acted as a supplementary part [152, 153].

## Conclusion

In this review, we concluded the current preclinical and clinical studies on BsAb against solid tumors, particularly anti-EpCAM, HER family, CEA, and PSMA. Taken together, the preclinical studies of BsAb showed potent antitumor efficacy, but the outcome of most clinical trials did not reach our expectation. In solid tumor, finding appropriate targets is the first step for the successful immunotherapy. The ideal target for BsAb would be the tumor specific antigens which are homogenously expressed on the surface of malignant cell and play a critical role in tumorigenesis. In spite, there are many antigens expressed in various tumor cells; it also needs to make great efforts to seek out more appropriate antigens to improve the specificity. The drawback of Fc-free BsAb is short half-life caused by its small molecular mass. To overcome the limitation, investigators developed several approaches including chemical coupling of polyethylene glycol (PEG) to the small molecule protein, fusion to heavy chain fragments (Fc/CH3) or HSA. Among these approaches, fusion of recombinant BsAb molecules to HSA significantly increased the circulation time but did not reduce the binding ability of recombinant BsAbs [154]. In addition, several obstacles have remained to be overcome for a successful application of BsAb in solid tumor, such as toxicity to normal tissues and low tumor/blood ratios. For example, compared with two-step method of pRIT, the three-step method significantly increased the tumor/blood ratios [133]. Based on the catumaxomab, blinatumomab, solitomab and other BsAbs, Trivedi et al. summarized the challenges of clinical pharmacology, pharmacometrics, and bioanalysis of BsAb and the possible solutions [155]. Through continuous efforts, investigators could find better approaches to overcome these challenges.

## Abbreviations

ADCC: Antibody-dependent cell mediated cytotoxicity
ADCP: Antibody-dependent cellular phagocytosis
AEs: Adverse events
ATC: Activated T cell
BiTEs: Bispecific T cell engagers
BsAb: Bispecific antibody
CA125: Cancer antigen-125;
CART: Chimeric antigen receptor T cell
CDC: Complement-dependent cytotoxicity
CEA: Carcinoembryonic antigen
CRC: Colorectal cancer
CRPC: Castrate resistant prostate cancer
CSCs: Cancer stem cells
DLT: Dose limiting toxicity
DOTA: 1, 4, 7, 10-tetraazacyclododecane-1, 4, 7, 10-tetraacetic acid
DT: Doubling time
DTPA: Diethylenetriaminepentaaceticacid
EGFR: Epidermal growth factor receptor
EOC: Epithelial ovarian cancer
EpCAM: Epithelial cell adhesion molecule
FDA: Food and Drug Administration
G-CSF: Granulocyte-colony stimulating factor
GM-CSF: Granulocyte-macrophage colony stimulating factor
GVHD: Graft-vs-host disease
HAHA: Human anti-human antibody
HAMA: Human anti-mouse antibody
HCC: Hepatocellular carcinoma
HER: Human epidermal growth factor receptor
HNSCC: Head and neck squamous-cell carcinoma
HSA: Human serum albumin
HSG: Histamine-succinyl-glycine
IGF-1R: Insulin-like growth factor-1 receptor
IL-2: Interleukin2
mAb: Monoclonal antibody
MTC: Medullary thyroid carcinoma
MTD: Maximum tolerated dose
NSCLC: Non-small-cell lung cancer
OS: Overall survival
PBMCs: Peripheral blood mononuclear cells
PD: Pharmacodynamics
PEG: Polyethylene glycol
PET: Positron-emission tomography
PFS: Progression-free survival
PK: Pharmacokinetics
pRIT: Pretargeting radioimmunotherapy
PSMA: Prostate-specific membrane antigen
RIT: Radioimmunotherapy
scFv: Single-chain variable fragments
SPECT: Single photon emission computed tomography
TAA: Tumor-associated antigen
TNFα: Tumor necrosis factor alpha
